# Phalangeal Fracture Secondary to Hammering One’s Finger

**DOI:** 10.7759/cureus.9313

**Published:** 2020-07-21

**Authors:** Sidhartha R Ramlatchan, Lauren H Pomerantz, Latha Ganti, Woo Kyung Lee, Gerald T Delk

**Affiliations:** 1 Emergency Medicine, Drexel University, Philadelphia, USA; 2 Medicine, University of Central Florida College of Medicine, Orlando, USA; 3 Emergency Medicine, Envision Physician Services, Nashville, USA; 4 Emergency Medicine, University of Central Florida College of Medicine, Orlando, USA; 5 Emergency Medical Services, Polk County Fire Rescue, Bartow, USA; 6 Emergency Medicine, Coliseum Medical Centers, Macon, USA

**Keywords:** phalangeal fractures

## Abstract

The authors report the case of a man who smashed his finger while using a hammer, resulting in a fracture-dislocation. The description of this injury type and the emergency management is discussed.

## Introduction

Normal hand anatomy encompasses four fingers, composed of three phalanges each, and one thumb, with two phalanges; these digits extend from the palm at the metacarpal bones forming a metacarpophalangeal (MCP) joint. Proximal (PIP) and distal interphalangeal (DIP) joints allow for further flexion and extension of each individual component of the digit. Via interosseous muscles and lumbricals shortening and lengthening, the flexor and extensor tendons can create these movements. When one or more components of the phalanx, such as the condyles, subcondyles, or shaft, break through the trabeculae and medullary cortex, it is considered fractured. Phalanx fractures can affect movement capabilities, resulting in unstable joints and malalignment. Various causes can result in a finger fracture, such as a rotational forces, hyperextension, or contact.

It is commonplace for finger fractures to heal well with nonsurgical management, resulting in few complications; however, certain locations of fractures are more prone to cause complications internally and require surgery. Fractures that occur in the proximal or middle phalanges, on some occasions, may drive the broken end of a bone into a joint which can damage its layer of cartilage [1]. This type of fracture in the proximal or middle phalanges is known as intra-articular because a joint is affected. If a joint is not affected by a fracture in the proximal or middle phalanges, the fracture is known as extra-articular. These fractures can be identified relatively easily by the deformity of the finger at the fracture site via x-ray or MRI [2]. Outside of the proximal or middle phalangeal fractures, fractures that occur in the distal phalanges are much less prone to complications, and are stable (meaning that the two broken ends stay aligned and do not interfere with any joints or protrude through the skin) [3]. The most common phalangeal fracture occurs at the distal phalanx. It easily heals with nonsurgical treatment such as a cast, splint, or closed reduction maneuver. Prevalence of fracture follows the pattern of distal>middle>proximal. For the index finger specifically, the overall prevalence of distal fracture is 39.7%, middle fracture is 33.3%, and proximal fracture is 27% [4].

Distal phalanx fractures can be subclassified into tuft, shaft, or intra-articular fractures, each based on the location within the affected bone. Tuft fractures are the most common and frequently result from crushing forces on the tip of the finger, resulting in painful swelling and shooting sensations [1]. Shaft fractures can most frequently occur in transverse and longitudinal planes, both of which are classified as stable and closed. Other types of fractures can transverse both planes such as spiral and oblique fractures. Intra-articular fractures associated with tendon avulsions (injury at the attachment site of the tendon to the bone) often present physically with a drooped fingertip due to lack of extension capabilities, specifically from the extensor tendon [5]. Middle phalanx fractures are the second most common location of phalanx fractures, and these similarly are subclassified via location involving the condylar, subcondylar, and shaft. Ecchymosis and swelling are the most pertinent physical exam findings. Proximal phalanx fractures are the least frequently observed due to the stabilizing support from collateral ligaments, extensor tendons, flexor tendons, and the volar plate ligament [6].

## Case presentation

A 21-year-old male patient presented to the emergency department (ED) immediately after he lacerated his right index finger while using a hammer. While attempting to hammer a nail, the patient accidently missed the nail and hit the proximal phalanx of the right index finger. He stated that all materials used during his labor, including the hammer and nail, had been clean at time of the incident. The patient reported no significant past medical history, past surgical history, family history, or social history.

Physical examination revealed that his right index finger was flexed at the PIP joint. Overlying the joint, there was a 1-cm laceration that was not actively bleeding at the time of examination. There were no other skin changes, redness, or swelling noted. There was no evidence of foreign body contamination. Evaluation of right arm pulses revealed intact radial pulse. The remainder of the physical exam including vital signs cardiovascular, pulmonary, abdominal, vascular, and neurological systems were unremarkable. 

**Figure 1:**
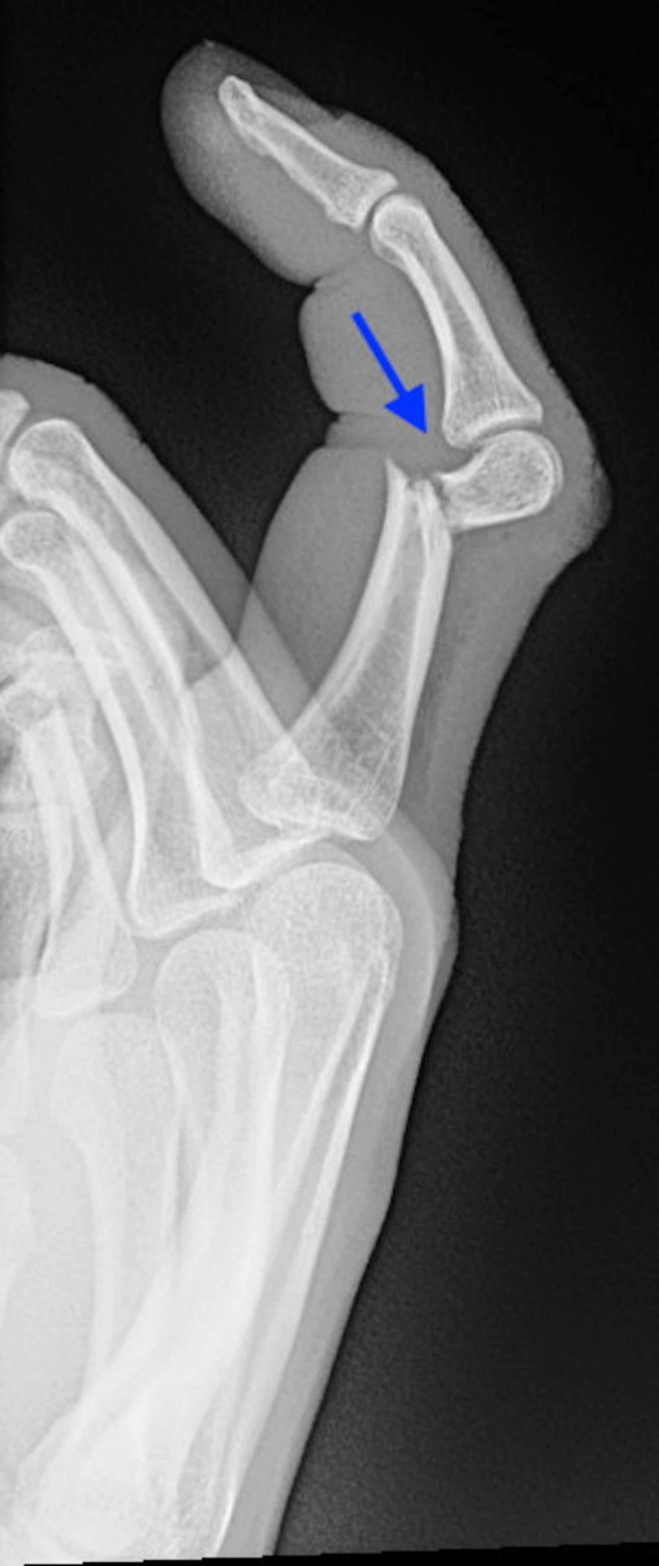
Lateral plain radiograph of the right index finger demonstrating fracture of the distal third of the proximal phalanx (arrow) with no bone-to-bone contact and intact articular surfaces

**Figure 2:**
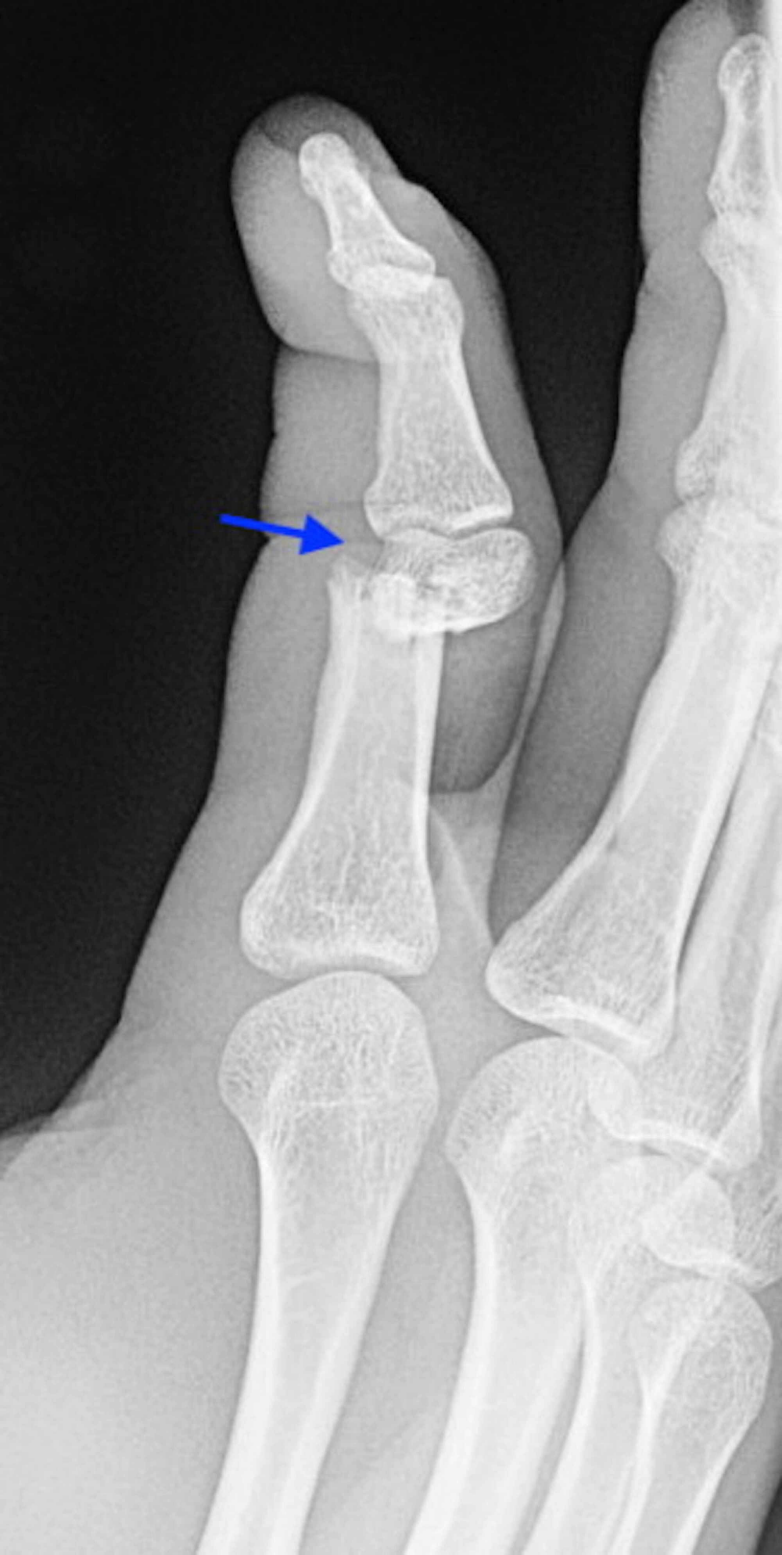
Oblique plain radiograph of the right index finger showing ventral dislocation (arrow) of the proximal metacarpal relative to the distal portion

**Figure 3:**
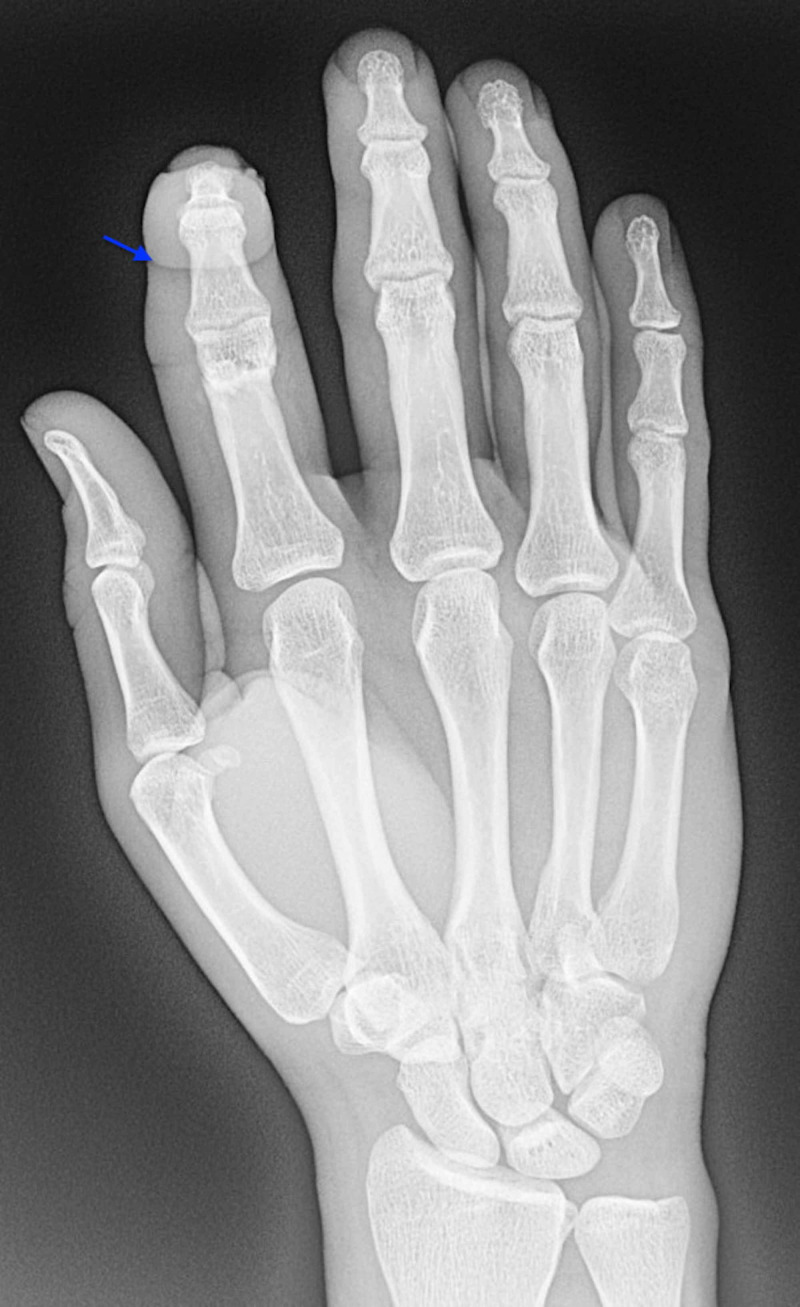
Posteroanterior plain radiograph of the right hand demonstrating flexion of the right index finger at the proximal interphalangeal joint

X-rays of the right index finger showed a significantly displaced fracture of the distal third of the proximal phalanx (Figures 1-3).

The fracture could be classified as a complete transverse fracture. This would be categorized as a type 3 phalangeal fracture due to displacement with lack of bone-to-bone contact [7]. The other fingers and thumb of the right hand were unaffected. The force of the hammer caused ventral dislocation of the proximal fragment and dorsal migration of the distal fragment with about 40 degrees of dorsal angulation. 

After the patient underwent the x-ray to identify the fracture, a hand surgeon was consulted and transfer to trauma surgery was recommended. Simultaneously, the wound was thoroughly irrigated with part povidone-iodine and part saline using an 18-gauge angiocath without the needle. The hand was meticulously cleansed including using soap and water. After proper irrigation and cleansing, the wound was dressed in sterile petroleum gauze and wrapped with sterile mesh gauze. A volar splint was placed. For analgesia, the patient received 0.5 mg intravenous (IV) hydromorphone. One gram of cefazolin IV was given for infection prophylaxis. The patient was transferred, taken to the operating room, and made a full recovery.

## Discussion

Phalanx fractures account for 10% of all fractures and are the most common fracture in the body [8]. Encompassing nearly 1.5% of all ED visits, phalanx fractures are common; however, it is imperative to understand that every injury to the finger is unique. Depending on the location of the fracture, and whether the fracture transverses through the entire bone or just part of it, such as an incomplete fracture, different treatment options are indicated. The importance of radiographs to determine the correct classification and treatment of the fracture is not to be understated [9]. Once classified, the physician can then appropriately determine possible treatment measures, as well as assess possible complications to be wary of.

Proximal phalangeal fractures of the index finger in adults, specifically, are the rarest form, and need to be critically analyzed to determine the best treatment plan moving forward. Subcondylar fractures tend to appear most commonly in children due to their open growth plates; however, in unique cases, accidents with contact force may produce enough force to yield a subcondylar break. Categorized into three types, subcondylar phalangeal fractures fall into one of the following: type 1 (displaced), type 2 (displaced with some bone to bone contact), or type 3 (displaced with no bone to bone contact) [10]. This variation presents with the option to perform a closed or open reduction. Closed reduction is the attempted alignment of bones without any surgical procedures in which force is applied to realign the affected bone fragments into the proper anatomical position via traction [11]. A splint is applied for protection and to keep the bones in the aligned position for healing [12]. Various splint types, ranging from long arm splints to volar splints to mallet splints, are indicated for different fractures that are mildly displaced and able to be manually healed with applied pressure. Closed reduction can also be achieved using external fixation, a technique in which bolts, pins, and metal components are used externally to align the bones in proper position. Metal components are drilled and inserted into the affected bones, and a frame is created using the components to hold the bones in the desired position. After the affected bones have healed correctly, the metal components are removed. It is important to periodically sanitize the areas of metal entry into the flesh to reduce the risk of infection. In comparison, an open reduction is a procedure that exposes a patient’s fractured bones and aims to correctly align them through surgical methods. Furthermore, internal fixation is achieved using hardware such as plates, screws, k-wires, or rods. Infection must also be monitored for due to the invasive nature of the procedure. Regardless of what type of reduction is performed, a radiograph of the fracture is taken post-procedure to ensure the reduction was successful.

Complications to monitor for following a phalangeal fracture include, but are not limited to, loss of motion, functional impairment, nerve damage, and atrophy following nonunion of the bone segments. Open reduction with internal fixation is the preferred method to minimize complications when the fracture is severe with displacement of the bone fragments being difficult to stabilize with nonoperative intervention.

## Conclusions

This case report presents a scenario in which a patient presented to the ED with flexion of his right index finger following an accident. Initial workup and radiography indicated a type 3 subcondylar phalangeal fracture that called for referral to trauma surgery.
